# Non-transmural myocardial infarction associated with regional contractile function is an independent predictor of positive outcome: an integrated approach to myocardial viability

**DOI:** 10.1186/s12968-021-00818-0

**Published:** 2021-11-01

**Authors:** Gianluca Di Bella, Giovanni Donato Aquaro, Jan Bogaert, Paolo Piaggi, Antonio Micari, Fausto Pizzino, Giovanni Camastra, Scipione Carerj, Mariapaola Campisi, Antonio Bracco, Maria Ludovica Carerj, Michele Emdin, Bijoy K. Khandheria, Alessandro Pingitore

**Affiliations:** 1 Clinical and Experimental Department of Medicine, University of Messina, via Consolare Valeria 1, 98100 Messina, Italy; 2 Fondazione Toscana G. Monasterio, via Giuseppe Moruzzi 1, 56124 Pisa, Italy; 3 Department of Radiology, KU Leuven – UZ Leuven, Gasthuisberg Campus. Herestraat 49, 3000 Leuven, Belgium; 4 Department of Information Engineering, University of Pisa, via G. Caruso 16, 56122 Pisa, Italy; 5 Department of Cardiology, “Santa Maria Dei Battuti” Hospital, Conegliano - ULSS2 Marca Trevigiana, Via Brigata Bisagno 2, 31015 Conegliano, Treviso Italy; 6 Cardiac Department, Vannini Hospital Rome, via Acqua Bullicante 4, 00177 Roma, Italy; 7 Aurora Cardiovascular and Thoracic Services, Advocate Aurora Health, Aurora Sinai/Aurora St. Luke’s Medical Centers, 2801 W. Kinnickinnic River Parkway, Ste. 880, Milwaukee, WI 53215 USA; 8 C.N.R. Clinical Physiology Institute, via Giuseppe Moruzzi 1, 56124 Pisa, Italy; 9 Present Address: Department of Cardiology, ISMETT” Hospital, via Ernesto Tricomi, 5, 90127 Palermo, Province of Palermo Italy

**Keywords:** Contractile segmental function, Myocardial infarction, Myocardial viability

## Abstract

**Background:**

Cardiovascular magnetic resonance permits assessment of irreversible myocardial fibrosis and contractile function in patients with previous myocardial infarction. We aimed to assess the prognostic value of myocardial fibrotic tissue with preserved/restored contractile activity.

**Methods:**

In 730 consecutive myocardial infarction patients (64 ± 11 years), we quantified left ventricular (LV) end-diastolic (EDV) and end-systolic (ESV) volumes, ejection fraction (EF), regional wall motion (WM) (1 normal, 2 hypokinetic, 3 akinetic, 4 dyskinetic), and WM score index (WMSI), and measured the transmural (1–50 and 51–100) and global extent of the infarct scar by late gadolinium enhancement (LGE). Contractile fibrotic (CT-F) segments were identified as those showing WM-1 and WM-2 with LGE ≤ or ≥ 50%.

**Results:**

During follow-up (median 2.5, range 1–4.7 years), cardiac events (cardiac death or appropriate implantable defibrillator shocks) occurred in 123 patients (17%). At univariate analysis, age, LVEDV, LVESV, LVEF, WMSI, extent of LGE, segments with transmural extent > 50%, and CT-F segments were associated with cardiac events. At multivariate analysis, age > 65 years, LVEF < 30%, WMSI > 1.7, and dilated LVEDV independently predicted cardiac events, while CT-F tissue was the only independent predictor of better outcome. After adjustment for LVEF < 30% and LVEDV dilatation, the presence of CT-F tissue was associated with good prognosis.

**Conclusions:**

In addition to CMR imaging parameters associated with adverse outcome (severe LV dysfunction, poor WM, and dilated EDV), the presence of fibrotic myocardium showing contractile activity in patients with previous myocardial infarction yields a beneficial effect on patient survival.

**Supplementary Information:**

The online version contains supplementary material available at 10.1186/s12968-021-00818-0.

## Background

Left ventricular (LV) dysfunction is a common finding after myocardial infarction (MI) that is associated with high morbidity and mortality in patients with heart failure [1, 2]. It is not only the extent of myocardial necrosis (subendocardial and/or transmural) and the severity of myocardial dysfunction in the jeopardized myocardium that contribute to post-MI ventricular remodeling, but other pathophysiological pathways (e.g., particularly neuroendocrine systems) involving the remote zones [3]. In this clinical context, contractile function represents a key element in counteracting post-ischemic remodeling and avoiding progression of LV dysfunction and heart failure [4].

Contractile function is synonymous with myocardial viability, although several definitions exist based on the markers used by different cardiac imaging techniques: wall thickness (echocardiography and cine cardiovascular magnetic resonance [CMR]), myocardial contractile reserve (dobutamine stress echocardiography/CMR), myocyte cellular integrity (single-photon emission computed tomography (SPECT)), myocardial metabolism (positron emission tomography (PET)), and myocardial scar/fibrosis (late gadolinium enhancement [LGE]) [4–9]. It is notable that CMR provides the opportunity to assess both the transmural extent of irreversible myocardial damage and contractile function. These two variables often are intermingled as they can coexist within the same myocardial segment, and, importantly, the likelihood of improvement in contractility after revascularization is predicted by the transmural extent of the scar, i.e., the lower the transmural extent of myocardial scar tissue, the higher the probability of functional recovery [10].

Therefore, although the clinical and prognostic impact of the different markers of myocardial viability have been extensively investigated, it is still unknown whether partially scarred segments showing contractile activity yield a prognostic value. The aim of this observational, multicenter study was to analyze the prognostic value of partially scarred myocardial segments yielding contractile activity and the potential additive impact in respect to the other predictors of adverse outcome in patients with previous MI.

## Methods

### Patients

We studied 862 consecutive patients with clinical evidence of a prior MI (> 90 days) and a clinical indication for CMR, i.e., quantification of LV function and volumes and identification of LV thrombi, extent of scar tissue, and myocardial viability. Previous MI was documented by clinical record, Q-waves at electrocardiography (ECG), and angiographic evidence of coronary artery stenosis (luminal diameter reduced by ≥ 50% in the left main stem and > 70% stenosis in a major coronary vessel). All patients were in stable clinical condition at enrollment. Patients were excluded if they had: (1) unstable angina or recent evidence of myocardial ischemia (absence of elevated troponin) (n = 57, 7%); (2) prosthetic annuloplasty and/or prosthetic heart valve (n = 24, 3%); (3) hypertrophic cardiomyopathy (n = 14, 2%); (4) history of malignancy and/or prior chemotherapy treatment (n = 16, 2%); (5) low-quality (non-diagnostic images) or incomplete CMR scan (n = 21, 3%). We also excluded patients with contraindications for CMR, irregular heart rhythm, and New York Heart Association class IV because of the difficulty of performing a diagnostic CMR scan. Eventually, 730 patients (85%) were included in the final stage of the study. Patients were studied either as outpatients (n = 160) or during hospitalization (n = 570). Clinical variables were collected before the CMR scan.

Institutional internal review boards of the involved hospitals approved our observational and retrospective study; the investigation complies with the principles outlined in the Declaration of Helsinki. All patients gave informed consent before being enrolled.

### CMR data acquisition

CMR was performed using a 1.5 T whole-body CMR scanner (CVi, HD release, GE Medical Systems, Milwaukee, Wisconsin, USA; Gyroscan NT, Philips Healthcare, Amsterdam, the Netherlands).

According to the protocols recommended by the Society for Cardiovascular Magnetic Resonance, a breath-hold  balanced steady-state free-precession ECG triggered sequence was used to evaluate global LV function. LGE images were obtained 10–20 min after a bolus injection of gadolinium-based contrast medium (0.1 mmol/kg of gadolinium-BOPTA, Multihance, Bracco, Milan, Italy; 0.2 mmol/kg of gadolinium-DOTA, Dotarem, Guerbet, Roissy, France; or 0.2 mmol/kg of gadobutrol, Gadovist, Bayer AG, Basel, Switzerland) using fast gradient-echocardiography inversion recovery sequences. Inversion time was individually adapted to null the signal of remote myocardium (usual range 220–300 ms). Cine and LGE images were acquired in at least one set of matched views including a contiguous LV short-axis and one vertical and one horizontal long-axis view.

### CMR data analysis

All CMR data were analyzed off-line using commercial software (Mass, Medis, Leiden, The Netherlands). CMR studies were centrally analyzed in a core-lab with consensus among 3 experienced observers (> 5 years’ experience) who were blinded to the clinical results and follow-up data.

Endocardial and epicardial LV contours were drawn on short-axis cine images at end-diastole and end-systole. Papillary muscle and trabeculations were considered part of the cavity. From these contours, LV end-diastolic volume (LVEDV), LV end-systolic volume (LVESV), LV ejection fraction (LVEF), and LV mass were calculated. Volumetric measures were normalized to body surface area [11]. A 17-segment model was used to describe LV segmental wall motion (WM). The WM of each segment was graded semiquantitatively (1 normal, 2 hypokinetic, 3 akinetic, 4 dyskinetic), and WM score index (WMSI) was then derived (sum of semiquantitative WM divided by 17).

**Fig. 1:**
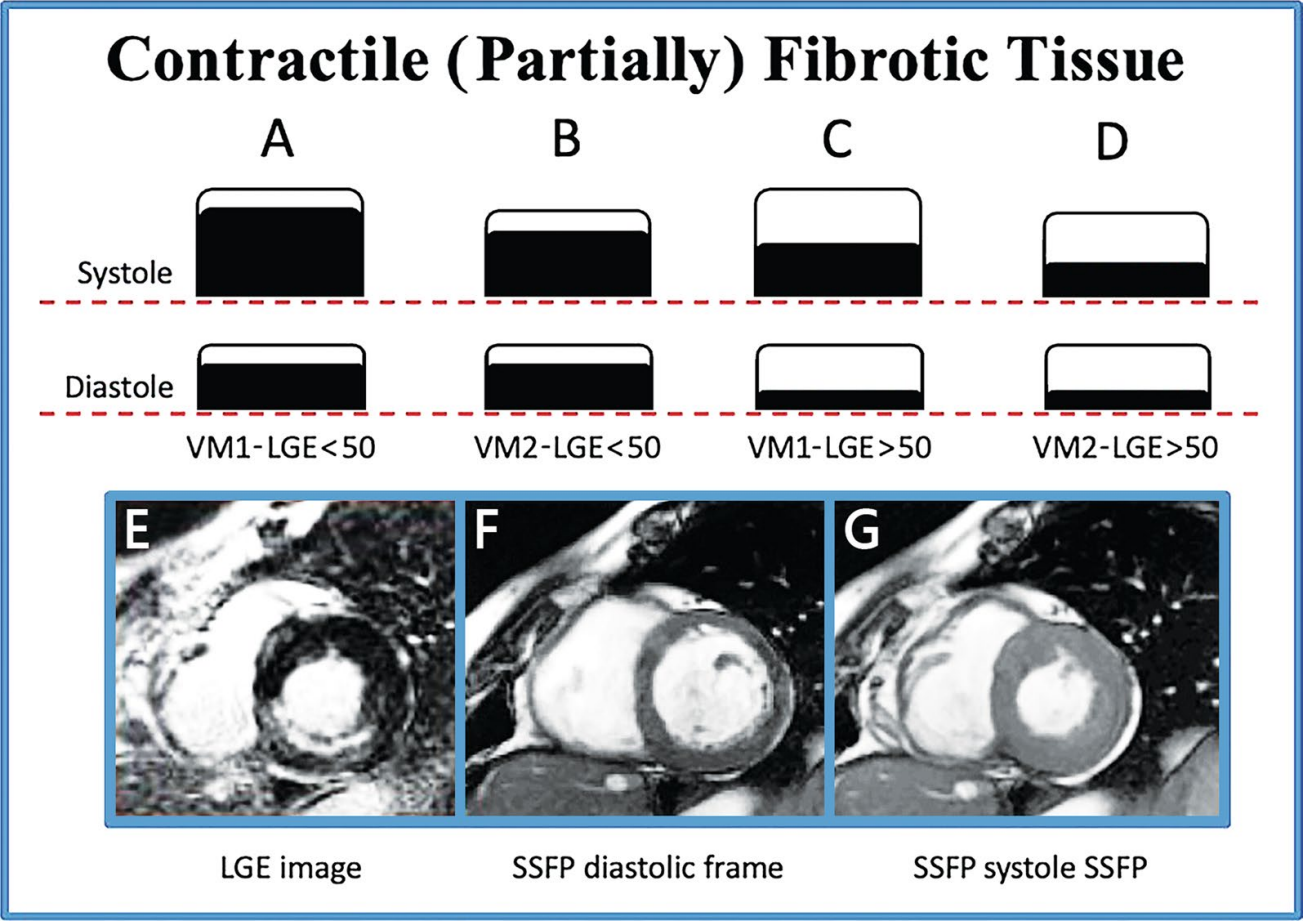
Example of illustration of contractile (partially) fibrotic (CT-F) segments. CT-F segments were identified as those showing wall motion (WM-1) and late gadolinium enhancement (LGE) < 50% (**A**), WM-2 and LGE < 50% (**B**), WM-1 and LGE > 50% (**C**), or WM-2 and LGE > 50% (**D**). Panels E–G show an example CT-F segment. Hyper-enhancement area (**E**, LGE < 50%) in basal inferior segment associated with a good contractility (WM1) comparing diastolic frame (**F**) and systolic frame (**G**) on cine cardiovascular magnetic resonance images. CT-F, contractile fibrotic; LGE, late gadolinium enhancement; SSFP, steady-state free precession; WM, wall motion

The total extent of myocardial LGE was determined semi-automatically, using a cut-off of 5 standard deviations (SD) above the average of remote myocardium. If needed, contours were manually corrected [12, 13]. The transmural extent of LGE was semiquantitatively measured in each segment and expressed as a percentage of the total thickness of the wall segment; it was subsequently clustered as segments with subendocardial (partially scarred) LGE (involving 1–50% of the segment wall thickness) and transmural (scarred) LGE (> 50% of the segment wall thickness) [10]. Next, the cine and LGE findings per segment were compared. Contractile fibrotic (CT-F) segments were defined as myocardial segments showing WM-1 and evidence of scarring (i.e., LGE ≤ 50% or LGE > 50%) and/or segments having WM-2 and LGE > 50% (Fig. 1). To assess intraobserver reproducibility, the same reader repeated this assessment in 100 cases with one week between readings; interobserver reproducibility was determined after a second experienced observer, blinded to the readings by the first observer, made an assessment.

### Follow-up

Follow-up was performed using a questionnaire administered to the physician during periodic ambulatory workup at our institutions (613 patients, 84%) or by telephone call (117 patients, 16%). This questionnaire was administered every year. Cardiac events (CEs) were defined as cardiac death and appropriate implantable cardioverter-defibrillator (ICD) shock. Appropriate ICD shock was considered equivalent to cardiac death. The cause of death was documented from medical records or death certificates. The definition of cardiac death required the documentation of life-threatening arrhythmia or cardiac arrest, or death attributable to heart failure or MI in the absence of any other precipitating factor. In the case of out-of-hospital death not followed by an autopsy, a sudden, unexpected death was considered cardiac death. ICD shocks were appropriate if triggered by malignant arrhythmia (ventricular tachycardia above the programmed cut-off of the ICD [12 intervals at 180 beats/min] or ventricular fibrillation). A complete interrogation of the ICD was performed by the referring physician in order to confirm the appropriateness of the shock [14].

Post-CMR coronary revascularization (either percutaneous or surgical) also was recorded. Coronary artery disease events included MI and post-CMR coronary revascularization.

### Statistical analysis

Data analyses were performed using SPSS (version 21, Statistical Package for the Social Sciences, International Business Machines, Inc., Armonk, New York, USA). Statistical significance was considered *P* < 0.05; all statistical tests were two-sided. Continuous variables were expressed as mean ± SD or median (25th; 75th percentiles) as appropriate; categorical variables were expressed as number and percentage. The comparison between continuous variables in patients with and without CEs was performed by Student’s independent samples t-test (Gaussian variables) or Wilcoxon test (skewed variables). The comparison between categorical variables was performed either by the chi-squared test or by the Fisher’s exact test if an expected cell count was < 5. The correlation between continuous variables was quantified by Pearson’s correlation coefficient.

The inter- and intraobserver analysis of CT-F segments was performed by calculating the intraclass correlation coefficient (ICC).

LVEDV and LVEF were dichotomized according to validated cut-offs. In post-hoc analysis, median value was used to discriminate large extent/involvement of WMSI (> 1.7), LGE extent (> 13%), and number of segments with LGE > 50% (≥ 3 segments).

Univariate Cox regression analysis was used to determine which variables were associated with CEs. In multivariate stepwise analysis using the forward selection technique based on the Wald statistic, all significant continuous or dichotomous variables at univariate analysis (*P* < 0.05) were included as potential predictors. Given that LVEDV and LVEF were strongly interrelated (r = −0.70), two multivariate models including either LVEF (model 1) or LVEDV (model 2) were computed separately; conversely, both WMSI and LGE were included in the same multivariate model as there was no substantial collinearity (tolerance > 0.6) that might have affected estimation.

On the basis of the global chi-squared statistic of each multivariate model, a risk score was computed based on the additive value of each significant dichotomous predictor in the final model. Specifically, each predictor contributed to the risk score with a weight proportional to the additive chi-squared statistic calculated in the stepwise analysis.

We used absence of CT-F (CT-F tissue not observed) instead of presence of CT-F to parallel the prognostic meaning of the other variables included in the risk score calculation.

The Kaplan–Meier survival function was used to represent the free-event survival in the population.

## Results

The median interval between MI and CMR was 7.5 months (IQR 3–80). Patients with CEs were older (*P* = 0.002), more likely to use diuretic and aldosterone receptor antagonist therapies (*P* < 0.001), and more likely to have > 1 MI (*P* = 0.005) (Table 1). The CE group had higher LV volumes and WMSI and lower LVEF (all *P* < 0.001). The amount of scarring was greater in the CE group (*P* = 0.003), and these patients had significantly more LGE segments, in particular, segments with LGE > 50% (*P* = 0.002).

**Table 1:** Clinical characteristics and cardiovascular magnetic resonance (CMR) variables in entire population and in subgroups with and without cardiac events

	Entire population (n = 730)	Cardiac events (n = 123)	No cardiac events (n = 607)	*P-*value
Age (years)	63 ± 12	66 ± 10	63 ± 12	0.002
Male sex (%)	88	87	88	0.349
Family history of CAD (%)	48	43	49	0.063
Hypertension (%)	58	59	58	0.151
Diabetes (%)	27	28	27	0.878
Hypercholesterolemia (%)	59	60	59	0.974
No. of stenosed vessels	1.8 ± 0.9	± 0.9	1.8 ± 0.9	0.09
Beta-blocker (%)	79	87	77	0.06
Diuretics (%)	46	69	40	< 0.001
ACE-I/ARB (%)	80	84	79	0.357
ANTI ALD (%)	30	46	26	< 0.001
> 1 MI (%)	16	25	14	0.005
ICD (%)	24	54	18	< 0.001
CMR data				
LVEDV (ml/m^2^)	111 ± 41	136 ± 46	106 ± 38	< 0.001
LVESV(ml/m^2^)	71 ± 42	99 ± 43	65 ± 39	< 0.001
LVEF (%)	41 ± 16	30 ± 10	43 ± 16	< 0.001
LV mass index	77 ± 22	82 ± 24	76 ± 21	0.02
LVEF < 30% (%)	29	55	24	< 0.001
Dilated LVEDVi	39	67	34	0.076
WMSI	1.7 ± 0.5	2.0 ± 0.5	1.7 ± 0.5	< 0.001
WMSI > 1.7 (%)	52	48	62	< 0.001
LGE extent (% of LV mass)	14 ± 8	16 ± 8	14 ± 8	0.003
LGE extent > median	50	62	47	0.003
Segments with LGE	6.0 ± 3.2	6.7 ± 3.4	5.9 ± 3.2	0.02
LGE transmural extent 1–50% (no. of segments)	2.2 ± 2.5	2.1 ± 2.4	2.2 ± 2.5	0.61
LGE transmural extent 51–100% (no. of segments)	3.8 ± 2.9	4.6 ± 3.1	3.7 ± 2.8	0.002
CT-F tissue (no. of segments)	1.9 ± 2	1.5 ± 1.7	2.0 ± 2.0	0.02

ACE-I, angiotensin converting enzyme inhibitor; aldosterone antagonists; ARB, angiotensin receptor blocker; CAD, coronary artery disease; CMR, cardiac magnetic resonance; CT-F, contractile fibrotic; ICD, implantable cardioverter defibrillator; LGE, late gadolinium enhancement; LV, left ventricular; LVEDV, left ventricular end-diastolic volume; LVEDVi, left ventricular end-diastolic volume indexed; LVEF, left ventricular ejection fraction; LVESV, left ventricular end-systolic volume; MI, myocardial infarction; WMSI, wall motion score index

During follow-up, a new coronary event was observed in 212 (29%) of 730 patients. Of these, 62/197 coronary events (31%) were in patients with absence of CT-F and 150/533 (28%) in patients with presence of CT-F (*P* = 0.33).

A total of 1429 of 12,410 segments (11%) were defined as CT-F. Of these segments, most were WM-1 and LGE < 50% (207 segments, 14%) or WM-2 and LGE < 50% (337 segments, 24%), 500 (35%) were WM-2 and LGE > 50%, and 385 (27%) were WM-1 and LGE > 50%. None of the segments in the last group had a transmural LGE extension of > 75%. On the contrary, WM-3 and -4 were associated with LGE < 50% in 1896 segments (15% of total segments) and with LGE > 50% in 578 segments (5% of total segments). The group without CEs had a higher number of CT-F segments at follow-up than the CE group (*P* = 0.02). There was strong intraobserver consistency (ICC = 0.93, 95% CI 0.91–0.94, *P* < 0.001**)** and interobserver consistency (ICC = 0.92, 95% CI 0.91–0.94, *P* < 0.001).

During follow-up (median 2.5 years, IQR 1.1 to 4.7), 123 patients (17%) had CEs (68 cardiac-related deaths and 55 appropriate ICD shocks).

### Predictors of cardiac death

At univariate analysis, age, LV volumes, LVEF, WMSI, extent of LGE, segments with transmural extent > 50%, and CT-F segments—both when considered as a continuous variable (Table 2) or as dichotomic variables (Table 3)—were associated with CEs.

**Table 2:** Hazard ratios of continuous variables for cardiac events (cardiac death and appropriate ICD shocks) at univariate and multivariate analysis in patients with previous myocardial infarction

Variable	Univariate	Multivariate model 1 (EF)	Multivariate model 2 (EDV)
Age (years)	1.028 (1.011–1.045)^a^	1.032 (1.015–1.050)^b^	1.028 (1.011–1.046)^a^
> 1 AMI	1.560 (0.991–2.456)		
MI to CMR interval	1.007 (0.970–1.045)		
Post-CMR coronary revascularization	1.225 (0.846–1.773)		
LVEDV ml/m^2^	1.009 (1.005–1.012)^b^	1.007 (1.003–1.011)^a^	
LVESV ml/m^2^	1.009 (1.006–1.012)^b^		
LVEF	0.954 (0.941–0.967)^b^		0.962 (0.946–0.978)^b^
LV Mass index	0.114 (0.998–1.016)		
WMSI	2.801 (1.949–4.024)^b^	2.024 (1.336–3.068)^a^	
No. segments with LGE	1.029 (0.977–1.084)		
No. segments with LGE 1–50%	0.934 (0.867–1.045)		
No. segments with LGE > 50%	1.087 (1.025–1.153)^a^		
LGE extent (% of LV mass)	1.025 (1.005–1.045)^c^		
CT-F	0.858 (0.772–0.955)^a^	0.479 (0.330–0.694)^b^	0.516 (0.357–0.747)^b^

Data presented as hazard ratio (95% CI)

^a^ < 0.01

^b^ < 0.001

^c^ < 0.05

**Table 3:** Hazard ratios of dichotomic variables for cardiac events (cardiac death and appropriate ICD shocks) at univariate and multivariate analysis in patients with previous myocardial infarction

Dichotomic variable	Univariate	Multivariate model 1 (LVEF < 30%)	Multivariate model 2 (LVEDV dilated)
Age > 65 years	1.65 (1.157–2.372)^a^	1.649 (1.149–2.367)^a^	1.664 (1.161–2.386)^a^
LVEF < 30% (cut-off for severe)	2.682 (1.878–3.829)^a^	1.956 (1.338–2.860)^a^	
Dilated LVEDV (> 112 ml/m^2^)	2.796 (1.916–4.080)^b^		2.047 (1.351–3.103)^a^
WMSI > 1.7 (median)	3.210 (2.094–4.919)^b^	2.235 (1.413–3.534)^a^	2.066 (1.289–3.310)^a^
LGE > 13% (median)	1.611 (1.120–2.319)^c^		
Segments with > 50% LGE (≥ 3 segments)	1.533 (1.075–2.185)^c^		
Presence of CT-F myocardium	0.476 (0.332–0.683)^b^	0.538 (0.374–0.774)^a^	0.516 (0.359–0.742)^b^

Data presented as hazard ratio (95% CI)

^a^ < 0.01

^b^ < 0.001

^c^ < 0.05

CT-F segments (chi-square 28.1) had an incremental predictive value (*P* < 0.001) compared to segments with transmural extent > 50% (chi-square 7.7). Also, at multivariate analysis, selected identical parameters—both when considered as continuous (Table 2) or dichotomic (Table 3) variables—were independent predictors of CEs. Particularly, age, LVEF, WMSI, and dilated LVEDV indexed were independent predictors of worse outcome, whereas the presence of CT-F myocardium was the only predictor of better outcome (Additional file 1: Tables S1–S3).

**Fig. 2:**
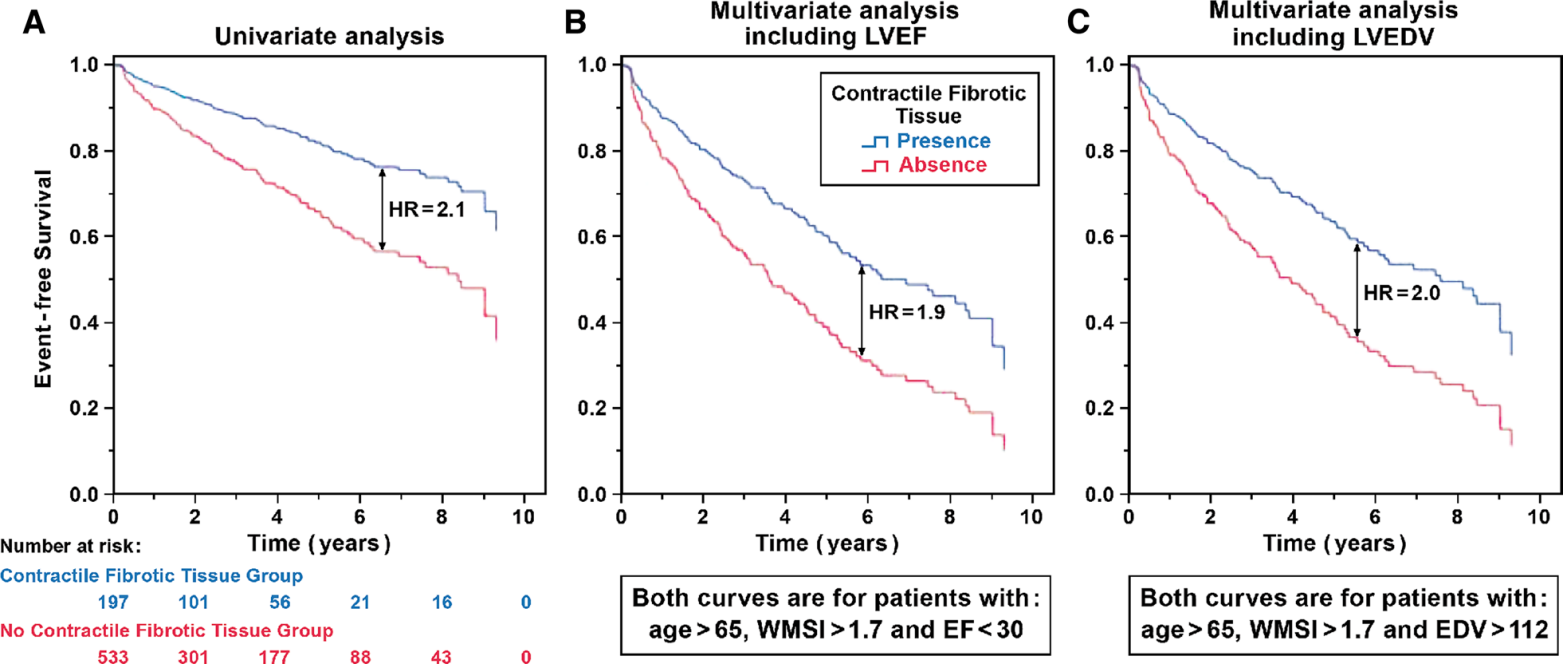
Kaplan–Meier survival curves according to presence of contractile fibrotic tissue in entire population (**A**) and in patients with high risk (age > 65, WMSI > 1.7) and LVEF < 30 or LVEDV > 112 (**B**, **C**, respectively). LVEDV, end-diastolic volume; LVEDV, left ventricular end-diastolic volume; LVEF, left ventricular ejection fraction; WMSI, wall motion score index

On survival analysis (Fig. 2A), patients with CT-F tissue (n = 535, 75 CEs) had better prognosis (HR for CEs = 0.48, 95% CI 0.33–0.68, *P* < 0.001) than those without (195 patients, 48 CEs).

Patients with the presence of CT-F myocardium had a higher cumulative survival at 2, 5, and 7 years than those without (Table 4).

**Table 4:** Survival at 2 years, 5 years, and 8 years according to CT-F

Follow-up (years)	Lack of CT-F myocardium (%)	Presence of CT-F myocardium (%)	Overall (%)
2	82.5	92.0	89.5
5	65.9	82.0	77.7
7	58.9	74.4	70.2

CT-F, contractile fibrotic

The estimated Kaplan–Meier survival curves in high-risk patients (age > 65 years, WMSI > 1.7, and LVEF < 30% [Fig. 2B] or LVEDV indexed > 112 ml/m^2^ [Fig. 2C]) showed that the presence of CT-F tissue is associated with a better prognosis than seen in those without CT-F.

**Fig. 3:**
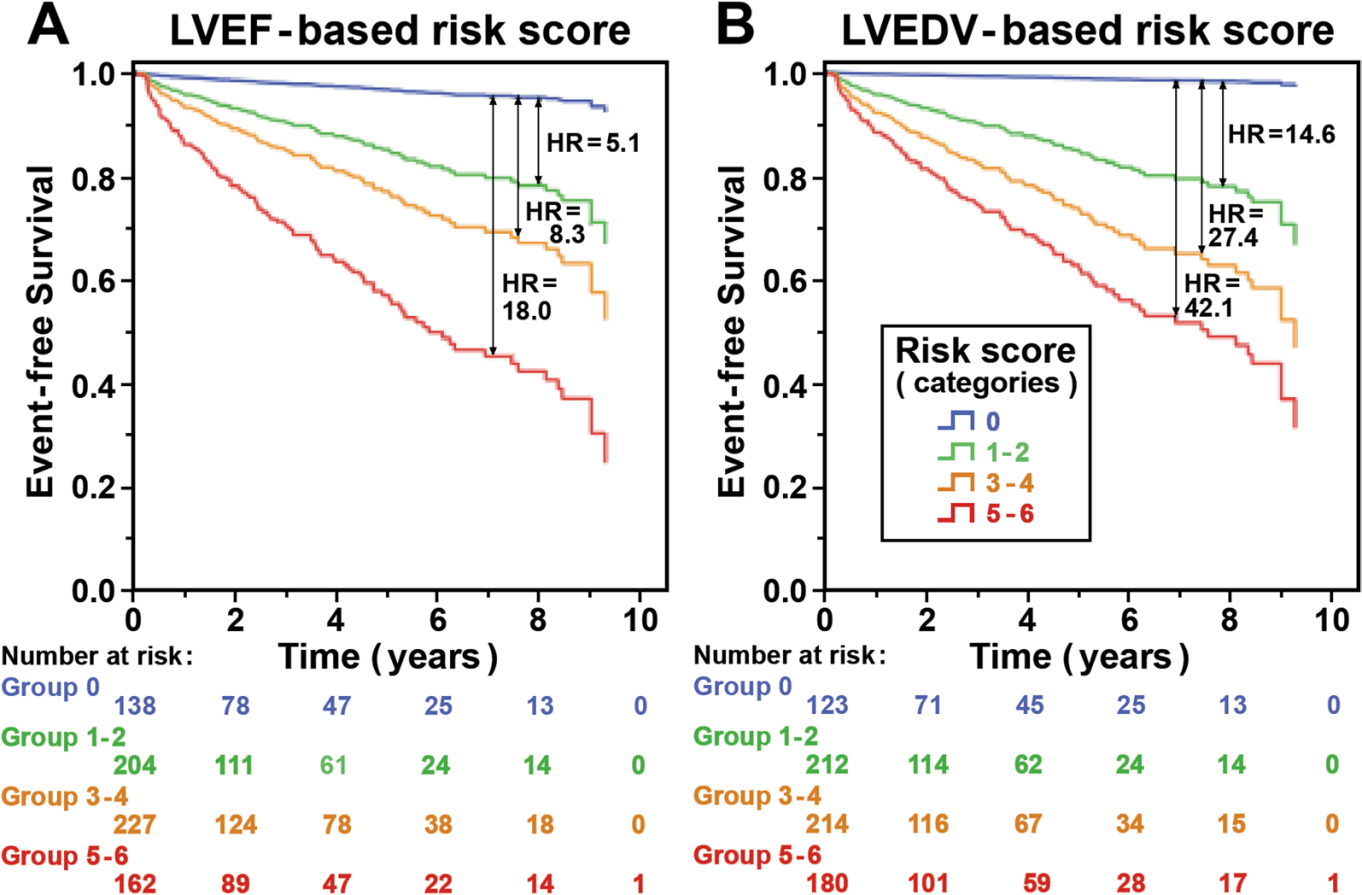
Kaplan–Meier survival curves according to six-unit risk score computed on stepwise Cox regression analyses generating groups. The four groups based on risk score—no risk (score 0, blue), low risk (score 1–2, green), moderate risk (score 3–4, orange), and high risk (score 5–6, red)—have considered left ventricular ejection fraction (**A**, LVEF) and end-diastolic volume (**B**, LVEDV), respectively

A six-unit risk score was computed based on the results of the stepwise Cox regression analyses. Specifically, three points were assigned to a WMSI > 1.7, one point to age > 65 years, one point to either an LVEF < 30% or a dilated LVEDV (> 112 ml/m^2^), and one point to the absence of CT-F. Subjects were then divided into four groups based on the values of their individual risk score, which allowed the identification of subsets of patients at no risk (score 0, blue), low risk (score 1–2, green), moderate risk (score 3–4, orange) and high risk (score 5–6, red) (Fig. 3, Additional file 1: Table S4). Figure 3 shows the Kaplan–Meier curves for the four categories of risk score, calculated with two models, one using LVEF (Fig. 3A) and the other LVEDV (Fig. 3B). As shown in Fig. 3, a significantly better event-free survival was observed in score 0 than in the other scores; similarly, a worse prognosis was observed in patients with a higher score in respect to those having a lower score (score 5–6 vs score 3–4; score 3–4 vs score 1–2).

## Discussion

The main finding of our study is that the presence of partially or fully preserved contractility in partially scarred myocardium in patients with previous MI is independently related to better outcome, with fewer CEs at follow-up. Also, in patients at high risk of cardiac death, including those > 65 years, high WMSI, and severe LV dysfunction (LVEF < 30%) or increased LVEDV, it has a positive prognostic value. The strength of this study is that we used comprehensive CMR to assess both WM and myocardial fibrosis, reflecting two faces of the same medal, i.e., myocardial viability. WM, as a viability marker, is used by echocardiography and CMR both at rest and after functional or pharmacological stress tests, whereas fibrosis, reflecting the amount of irreversibly damaged myocardium, is typically shown by LGE CMR [5]. The various approaches to predicting functional recovery have differing predictive value, with markers of contractility having the highest positive predictive value for prediction of functional recovery [15, 16]. To appreciate the degree of scar transmurality, we used LGE CMR. In the landmark paper by Kim et al., 78% of dysfunctional segments with no scar at LGE improved contractility after revascularization compared with 55% of those with < 50% transmural scar extent and only 2% of the segments with > 75% transmural scar extent [10]. Therefore, a cut-off of 50% of transmural extent of scar tissue is actually one of the CMR criteria of myocardial viability [5, 17–20].

Indeed, using this CMR definition of myocardial viability, Pegg et al. showed that the amount of viable and normal tissue (≥ 10 segments) was predictive of functional improvement after revascularization [20].

More recently, Kwon et al. used this parameter to define jeopardized myocardial segments, concluding that these segments were predictive of mortality when revascularization was not performed [19]. In contrast to the above-mentioned studies, in which the CMR criterion of myocardial segmental viability was based on the presence of < 50% transmural extent of fibrosis only, we added the evaluation of resting contractility in the same segment. However, because we assessed the patients in the chronic phase of post-MI, no predictions can be made with regard to contractility status immediately after acute MI. Thus, dysfunctional segments in the acute phase may be able to recover substantially after effective revascularization and/or medical therapy.

Prediction of functional recovery in the presence or absence of necrosis was not the goal of this study. In fact, the true novelty of our study was the assessment of the prognostic role of the combined presence of fibrosis and contractility in the same segment.

This is important because segments with non-transmural extent of fibrosis, according to the CMR criteria, are not a homogenous group since functional recovery occurs in a portion of them. Indeed, in the study of Pegg et al., about 63% of segments with < 50% transmural extent of necrosis had recovered function [20].

In regard to the results of our study, the presence of contractility in the partially scarred segments highlights the importance of segmental function in the prognostic stratification of patients with previous MI. This result confirms previous findings showing the independent prognostic role of WMSI in the prediction of CEs in patients with previous MI [14, 21–24].

In our study, the weight of segmental function/fibrosis was confirmed when CT-F tissue was included in the risk score together with age and other stronger prognostic CMR data, such as severe LV dysfunction and dilated LV.

The improvement of LV regional systolic function is a complex and dynamic phenomenon observed not only after coronary revascularization but also due to many medications [25–29]. Losartan and captopril (echocardiographic sub-study of OPTIMAAL trial) have been shown to be related to an improvement of segmental systolic function detected by a reduced WMSI [25]. As documented in the ANZ carvedilol trial, carvedilol therapy for one year was associated with reduced LV volumes, increased LVEF, and prevented progressive LV dilation in patients with heart failure due to ischemic heart disease [26]. The positive prognostic weight may be linked to several factors including improvement in global LV function [25], favoring reverse remodeling [30] and reducing the risk of arrhythmias [31]. All of these potential mechanisms may be responsible for the positive prognostic role of residual systolic function in post-MI patients. Further, it is intriguing that the positive prognostic weight of contractility (WM-1, -2) counteracts the negative one of myocardial fibrosis, i.e., function is a stronger prognostic variable than fibrosis when they are together in the same myocardial segments. This may account for the evidence that global extent of scar tissue loses its power to predict CEs when added to dilated LVEDV, severely depressed LVEF, and severe impairment of WMSI.

## Limitations

A major limitation of our study is that we do not have data about WM and LGE during the acute phase of MI, and thus we did not assess the possible contractile recovery of the partially fibrotic segments. However, based on previous studies, we supposed that CT-F tissue had a transient and reversible dysfunction due to necrosis and a successive improvement in WM due to acute or chronic revascularization and/or pharmacological data [10, 15, 16, 18, 25–29].

Neither the number of CT-F segments per patient nor the different CT-F patterns per each patient were assessed in terms of prognostic weight. Further studies are needed to show the potential prognostic impact of both the number of CT-F segments and the different CT-F patterns.

We did not measure systolic wall thickening. However, we used wall motion analysis, which is a well-defined and accepted semiquantitative method to assess regional systolic function in daily clinical practice, with the aim to assess the prognostic impact of variables used in this context. Also, we did not use a T1 mapping sequence because several examinations were dated and, thus, this sequence was not available. Further, this was a multicenter study using CMR units from different vendors, which would make comparison of T1 mapping data difficult. Moreover, we used semiquantitative methods to assess both WM and transmural extent of LGE. However, one of the objects of this study was to assess the current impact of CMR in daily clinical practice, where LGE technique and WM analysis are largely applied in the context of diagnostic and therapeutic decision-making; in opposition, T1 mapping and quantitative methods of LGE extent and contractility are actually outside the clinical scenario.

## Conclusions

In patients with previous MI, cardiac death is the result of multifactorial elements including clinical, bio-humoral, electrocardiographic, and several imaging variables. In the context of imaging parameters, in addition to adverse prognostic factors (severe LV dysfunction, high WM abnormalities, and dilated LVEDV), the presence of fibrotic segments having contractile activity had an independent protective effect on survival of patients with previous MI. Larger studies are needed to allow generation of a multiparametric score for risk stratification of these patients.

## Supplementary Information

**Additional file 1: Table S1.** Stepwise analyses, including the C-statistics at each step. **Table S2.** Hazard ratios of continuous variables for cardiac events (cardiac death and appropriate ICD shocks) within 5 years of follow-up at multivariate analysis in patients with previous MI. **Table S3.** Hazard ratios of dichotomic variables for cardiac events (cardiac death and appropriate ICD shocks) within 5 years of follow-up at multivariate analysis in patients with previous MI. **Table S4.** Risk score data distribution (range: 0–6) by 4 steps to risk score category with adequate sample size.

## Data Availability

The datasets used and/or analyzed during the current study are available from the corresponding author upon reasonable request.

## Abbreviations

CE: Cardiac event
CMR: Cardiovascular magnetic resonance
CT-F: Contractile fibrotic
ECG: Electrocardiogram
EDV: End-diastolic volume
EF: Ejection fraction
ESV: End-systolic volume
ICC: Intraclass correlation coefficient
ICD: Implantable cardioverter-defibrillator
LGE: Late gadolinium enhancement
LV: Left ventricle/left ventricular
LVEDV: Left ventricular end-diastolic volume
LVEF: Left ventricular ejection fraction
LVESV: Left ventricular end-systolic volume
MI: Myocardial infarction
SD: Standard deviation
WM: Wall motion
WMSI: Wall motion score index
