# Supplementary material for: Non-transmural myocardial infarction associated with regional contractile function is an independent predictor of positive outcome: an integrated approach to myocardial viability
Source: J Cardiovasc Magn Reson. 2021 Nov 1;23:121. doi: 10.1186/s12968-021-00818-0 (PMC8559354; doi:10.1186/s12968-021-00818-0)
Supplement: Supplementary file 1 — Additional file 1: Table S1. Stepwise analyses, including the C-statistics at each step. Table S2. Hazard ratios of continuous variables for cardiac events (cardiac death and appropriate ICD shocks) within 5 years of follow-up at multivariate analysis in patients with previous MI. Table S3. Hazard ratios of dichotomic variables for cardiac events (cardiac death and appropriate ICD shocks) within 5 years of follow-up at multivariate analysis in patients with previous MI. Table S4. Risk score data distribution (range: 0–6) by 4 steps to risk score category with adequate sample size. [file 12968_2021_818_MOESM1_ESM.docx]

**Additional file 1**

**Non-transmural myocardial infarction associated with regional contractile function is an independent predictor of positive outcome: An integrated approach to myocardial viability**

Gianluca Di Bella, Giovanni Donato Aquaro, Jan Bogaert, Paolo Piaggi, Antonio Micari, Fausto Pizzino, Giovanni Camastra, Scipione Carerj, Mariapaola Campisi, Antonio Bracco, Maria Ludovica Carerj, Michele Emdin, Bijoy K. Khandheria, Alessandro Pingitore

**Table S1 Stepwise analyses, including the C-statistics at each step**

| **Step** | **Multivariate Model 1 (EF)** | | | | **Multivariate Model 2 (EDV)** | | | |
| --- | --- | --- | --- | --- | --- | --- | --- | --- |
|  | **chi-square** | **df** | **sig.** | **C-stat** | **chi-square** | **df** | **sig.** | **C-stat** |
| 1 | 32.118 | 1 | <0.001 | 0.655 | 32.118 | 1 | <0.001 | 0.655 |
| 2 | 46.327 | 2 | <0.001 | 0.707 | 43.647 | 2 | <0.001 | 0.689 |
| 3 | 57.394 | 3 | <0.001 | 0.728 | 54.831 | 3 | <0.001 | 0.729 |
| 4 | 63.882 | 4 | <0.001 | 0.738 | 61.951 | 4 | <0.001 | 0.738 |

EDV, end-diastolic volume; EF, ejection fraction.

**Table S2 Hazard ratios of continuous variables for cardiac events (cardiac death and appropriate ICD shocks) within 5 years of follow-up at multivariate analysis in patients with previous MI**

| **Variable** | **Multivariate Model 1 (EF)** | Multivariate Model 2 (EDV) |
| --- | --- | --- |
| Age (years) | 1.031 (1.012-1.050)^b^ | 1.026 (1.006-1.046)^a^ |
| LV-EDV ml/m2 | 1.006 (1.001 - 1.010)^a^ |  |
| LV-ESV ml/m2 |  |  |
| LVEF |  | 0.963 (0.944-0.981)^b^ |
| WMSI | 2.488 (1.482 - 4.179)^b^ | n.s. |
| CT-F | 0.592 (0.396-0.886)^a^ | 0.603 (0.403-0.902) ^a^ |

Data presented as hazard ratio (95% CI).

^a^ ≤0.01

^b^ ≤0.001

n.s. >0.05

CT-F, contractile fibrotic segments; ICD, implantable cardioverter-defibrillator; EDV, end-diastolic volume; EF, ejection fraction; ESV, end-systolic volume; LV, left ventricular; MI, myocardial infarction; WMSI, wall motion score index.

**Table S3 Hazard ratios of dichotomic variables for cardiac events (cardiac death and appropriate ICD shocks) within 5 years of follow-up at multivariate analysis in patients with previous MI**

| **Dichotomic variable** | **Multivariate model 1 (LVEF<30%)** | Multivariate Model 2 (LV-EDV dilated) |
| --- | --- | --- |
| Age >65 years | n.s. | n.s. |
| LVEF <30% (cut-off for severe) | 2.234 (1.441-3.465)^a^ |  |
| Dilated LV-EDV (>112 ml/m^2^) |  | 1.904 (1.202-3.016)^a^ |
| WMSI >1.7 (median) | 1.928 (1.159-3.206)^a^ | 2.038 (1.223-3.395)^a^ |
| Presence of CT-F myocardium | 0.583 (0.393-0.864)^a^ | 0.587 (0.395-0.871)^a^ |

Data presented as hazard ratio (95% CI).

^a^ ≤0.01

n.s. >0.05

CT-F, contractile fibrotic segments; ICD, implantable cardioverter-defibrillator; EDV, end-diastolic volume; EF, ejection fraction; LV, left ventricular; MI, myocardial infarction; WMSI, wall motion score index.

**Table S4 Risk score data distribution (range: 0-6) by 4 steps to risk score category with adequate sample size**

|  | **Multivariate Model 1 (EF)** | **Multivariate Model 2 (EDV)** |
| --- | --- | --- |
| **Score bins** | **Frequency** | **Frequency** |
| 0 | 136 | 123 |
| 1-2 | 204 | 212 |
| 3-4 | 227 | 214 |
| 5-6 | 162 | 180 |

EDV, end-diastolic volume; EF, ejection fraction.
